# Supplementary material for: High-Strength, Self-Healing Copolymers of Acrylamide and Acrylic Acid with Co(II), Ni(II), and Cu(II) Complexes of 4′-Phenyl-2,2′:6′,2″-terpyridine: Preparation, Structure, Properties, and Autonomous and pH-Triggered Healing
Source: Polymers (Basel). 2024 Nov 9;16(22):3127. doi: 10.3390/polym16223127 (PMC11598322; doi:10.3390/polym16223127)
Supplement: Supplementary file 1 [file polymers-16-03127-s001.zip › polymers-3293874-supplementary.pdf]

polymers

Article

High-Strength, Self-Healing Copolymers of Acrylamide and Acrylic Acid with Co(II), Ni(II), and Cu(II) Complexes of 4′-Phenyl-2,2′:6′,2″-terpyridine: Preparation, Structure, Properties, and Autonomous and pH-Triggered Healing

Evgeny S. Sorin [1,*], Rose K. Baimuratova [1], Mikhail V. Zhidkov [1], Maria L. Bubnova [1], Evgeniya O. Perepelitsina [1], Ainur F. Abukaev [1], Denis V. Anokhin [1,2], Dmitry A. Ivanov [1,2,3] and Gulzhian I. Dzhardimalieva [1,4,*]

[1] Federal Research Centre of Problems of Chemical Physics and Medicinal Chemistry RAS, 142432 Chernogolovka, Russia; rozab@icp.ac.ru (R.K.B.); zhidkov@icp.ac.ru (M.V.Z.); bml@icp.ac.ru (M.L.B.); jane@icp.ac.ru (E.O.P.); ainurabukaev@gmail.com (A.F.A.); anokhin@icp.ac.ru (D.V.A.); dimitri.ivanov.2014@gmail.com (D.A.I.)
[2] Department of Chemistry, Lomonosov Moscow State University, 119234 Moscow, Russia
[3] Institut de Sciences des Matériaux de Mulhouse (CNRS UMR 7361), 68057 Mulhouse, France
[4] Moscow Aviation Institute, National Research University, 125993 Moscow, Russia
* Correspondence: sorin_es@mail.ru (E.S.S.); dzhardim@icp.ac.ru (G.I.D.); Tel.: +7-(496)-522-7763 (G.I.D.)

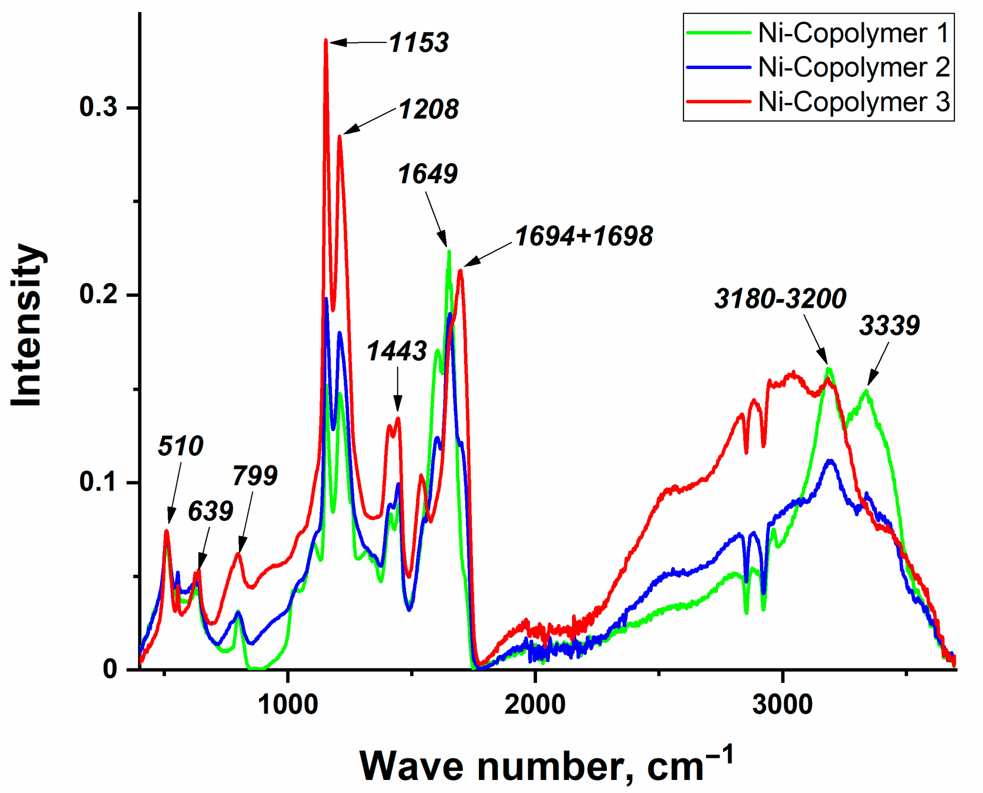

Figure S1. IR spectra of copolymers with 1 wt.% NiAcr$_2$PhTpy: Ni-Copolymer 1—AAm/AAc 84.5/14.5; Ni-Copolymer 2—AAm/AAc = 49.5/49.5; Ni-Copolymer 3—AAm/AAc = 14.5/84.5.

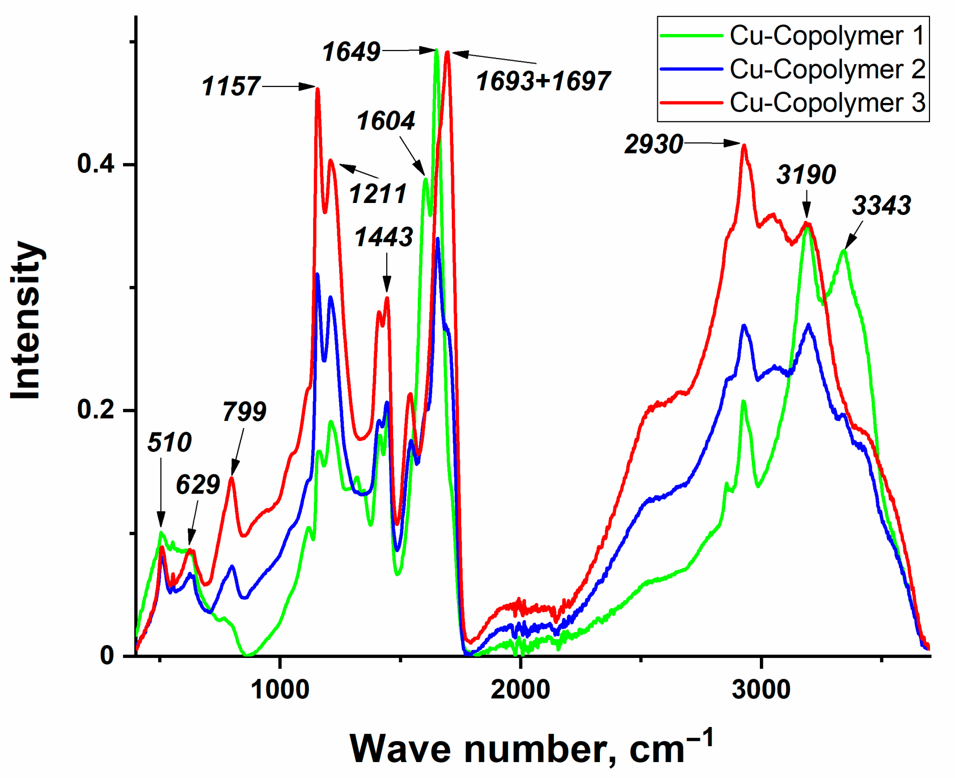

Figure S2. IR spectra of copolymers with 1 wt.% CuAcr2PhTpy: Ni-Copolymer 1—AAm/AAc 84.5/14.5; Cu-Copolymer 2—AAm/AAc = 49.5/49.5; Cu-Copolymer 3—AAm/AAc = 14.5/84.5.

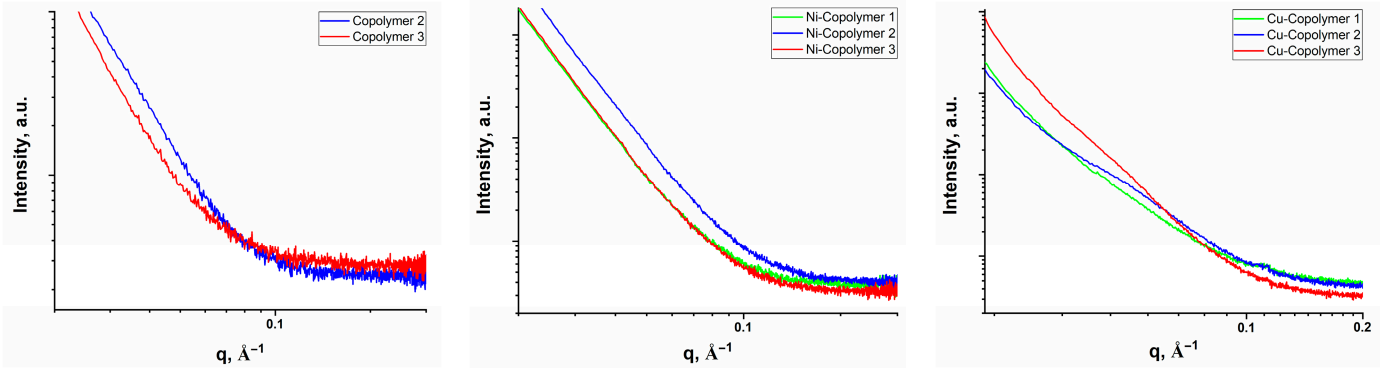

(a) **(b)** **(c)**

Figure S3. SAXS curves of (**a**) model copolymers (Copolymer 2—AAm/AAc = 50/50; Copolymer 3—AAm/AAc = 15/85) and copolymers with (**b**) 1 wt.% NiAcr2PhTpy and (**c**) 1 wt.% CuAcr2PhTpy (monomer ratios for both (**b**) and (**c**) systems: copolymer 1—AAm/AAc 84.5/14.5; copolymer 2—AAm/AAc = 49.5/49.5; copolymer 3—AAm/AAc = 14.5/84.5).

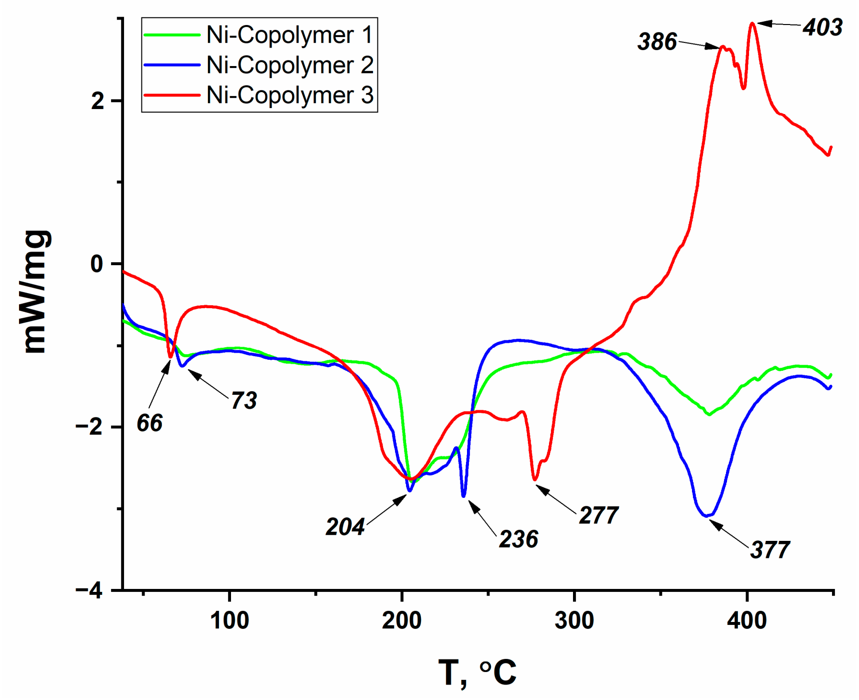

Figure S4. DCS curves of copolymers with 1 wt.% NiAcr2PhTpy: Ni-Copolymer 1—AAm/AAc 84.5/14.5; Ni-Copolymer 2—AAm/AAc = 49.5/49.5; Ni-Copolymer 3—AAm/AAc = 14.5/84.5.

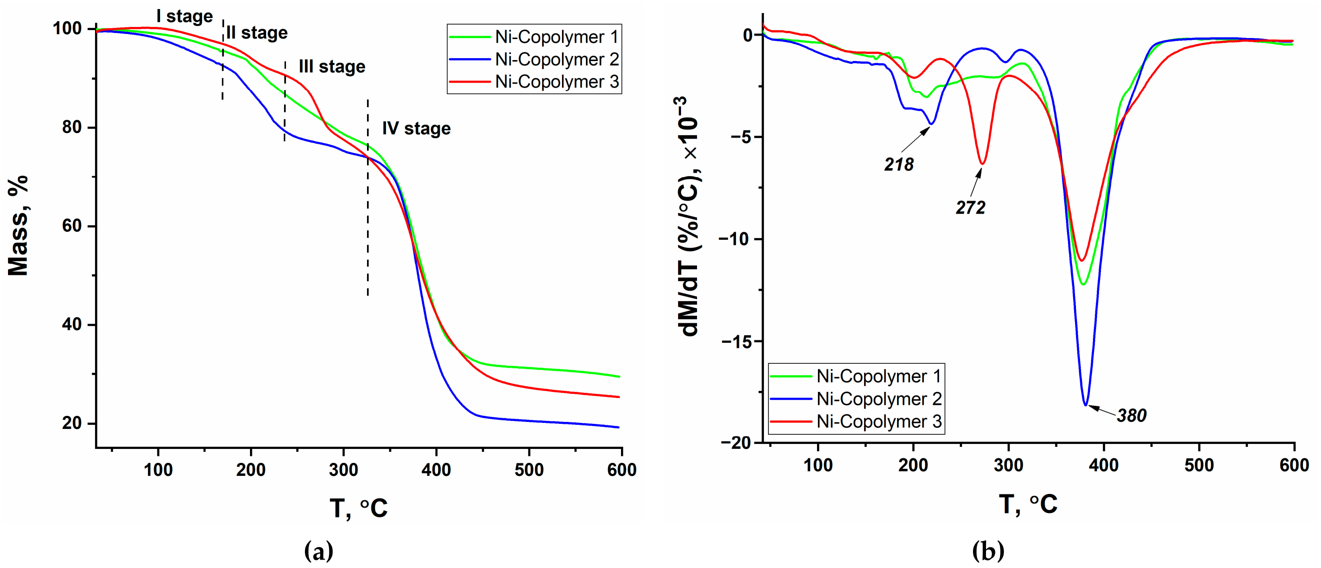

Figure S5. TGA (**a**) and DTG (**b**) curves of copolymers with 1 wt.% NiAcr2PhTpy: Cu-Copolymer 1—AAm/AAc 84.5/14.5; Cu-Copolymer 2—AAm/AAc = 49.5/49.5; Cu-Copolymer 3—AAm/AAc = 14.5/84.5.

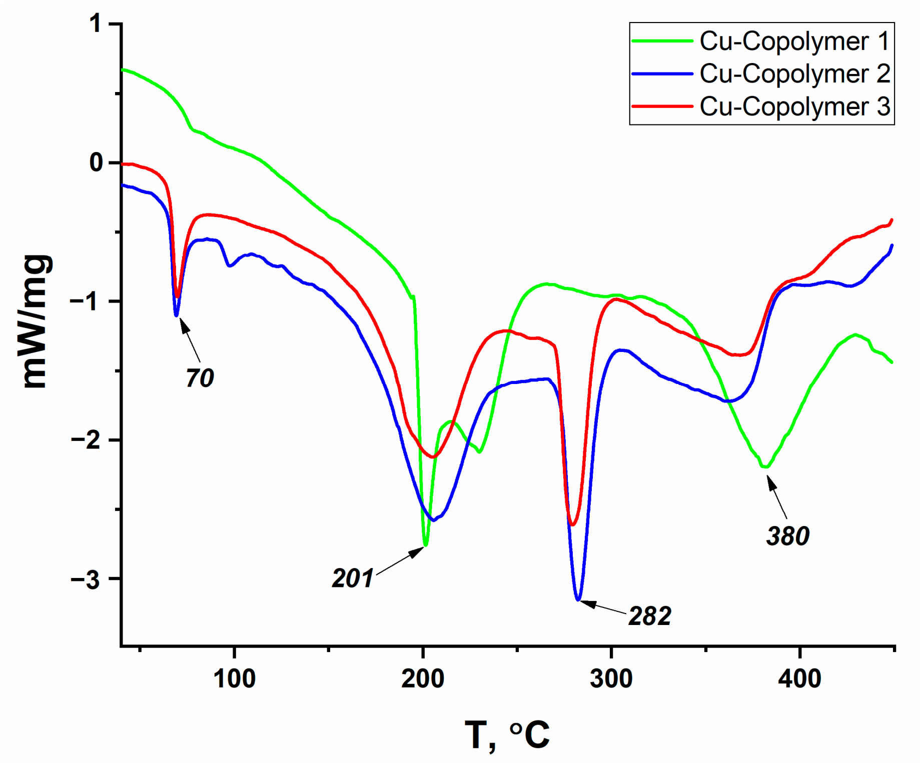

Figure S6. DCS curves of copolymers with 1 wt.% $CuAcr_2PhTpy$: Cu-Copolymer 1—AAm/AAc 84.5/14.5; Cu-Copolymer 2—AAm/AAc = 49.5/49.5; Cu-Copolymer 3—AAm/AAc = 14.5/84.5.

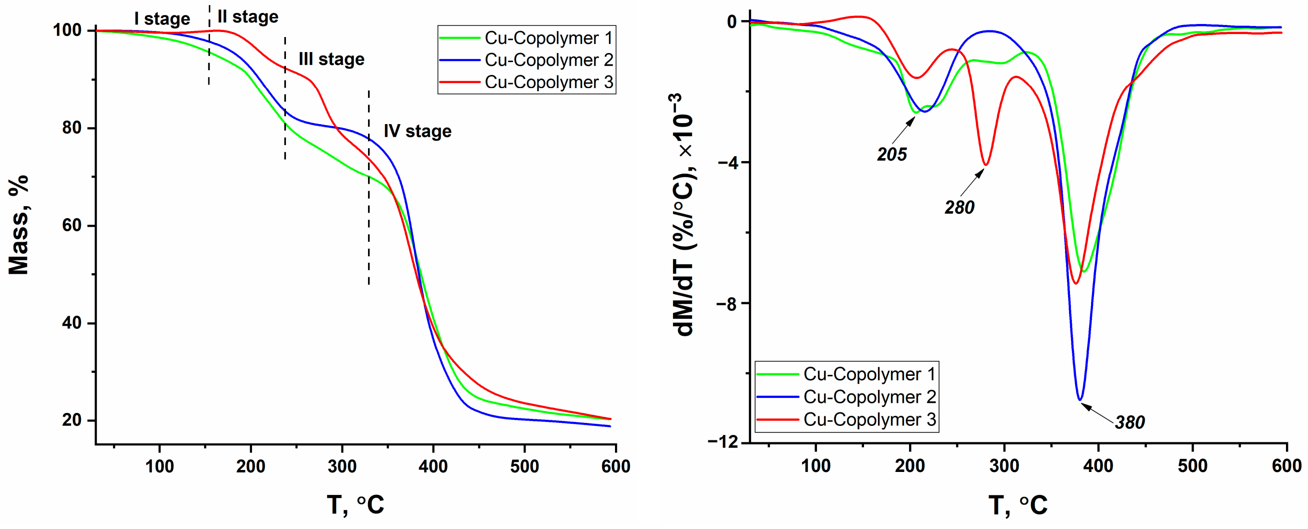

(a) (b)

Figure S7. TGA (**a**) and DTG (**b**) curves of copolymers with 1 wt.% $CuAcr_2PhTpy$: Cu-Copolymer 1—AAm/AAc 84.5/14.5; Cu-Copolymer 2—AAm/AAc = 49.5/49.5; Cu-Copolymer 3—AAm/AAc = 14.5/84.5.

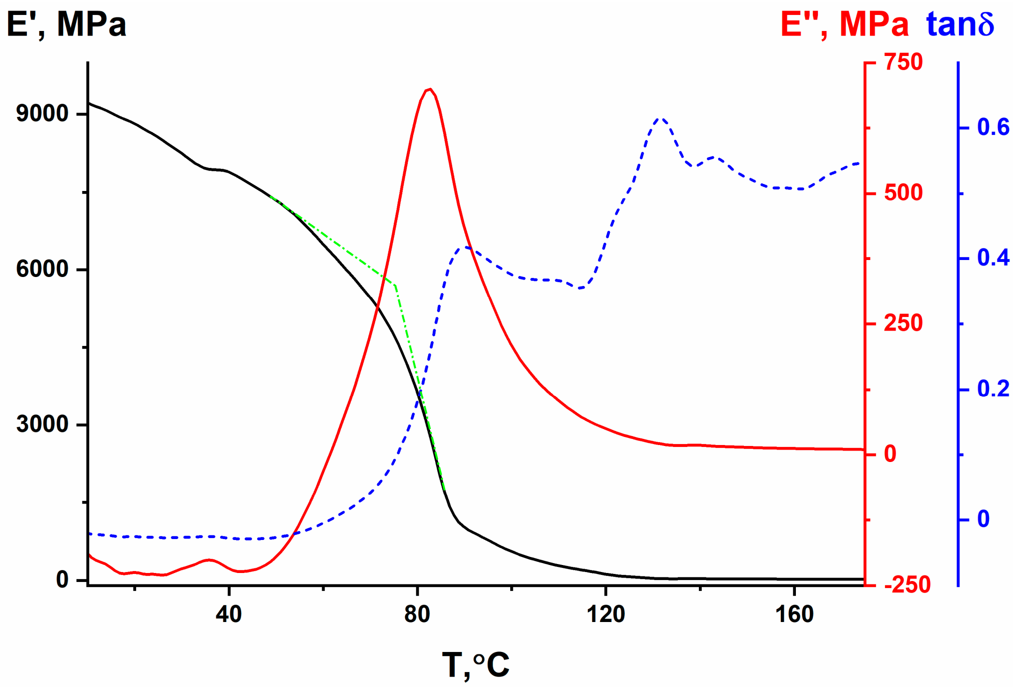

Figure S8. Dynamic mechanical analysis of Co-Copolymer 1: black line – elastic modulus, red line – loss modulus, blue dash – loss tangent, green dash-dot – tangents for glass transition temperature determination.

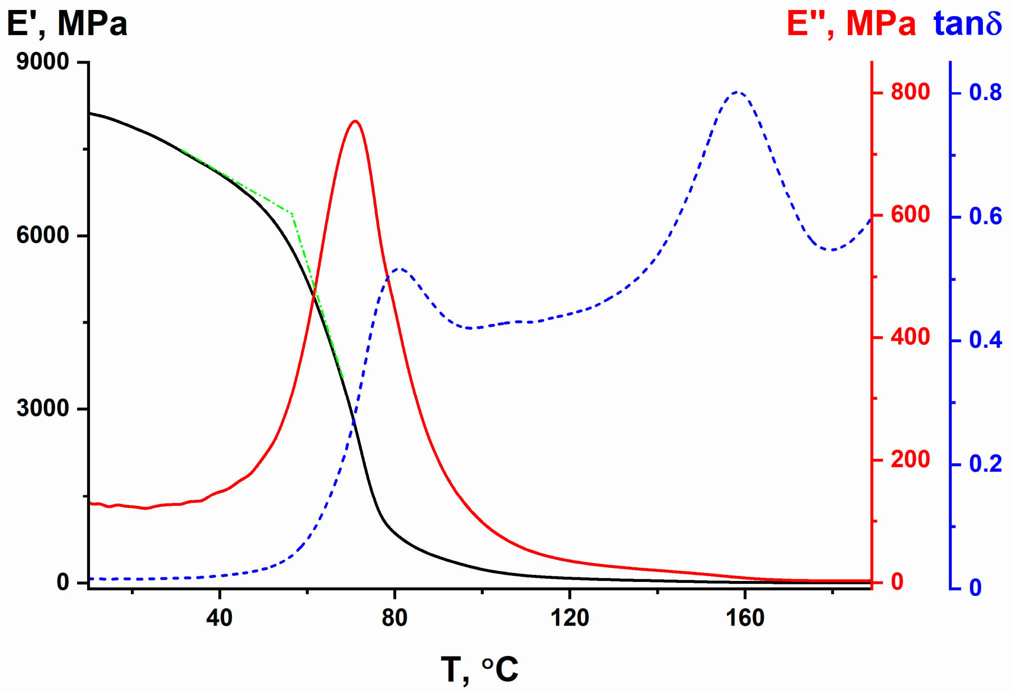

Figure S9. Dynamic mechanical analysis of Co-Copolymer 2: black line – elastic modulus, red line – loss modulus, blue dash – loss tangent, green dash-dot – tangents for glass transition temperature determination.

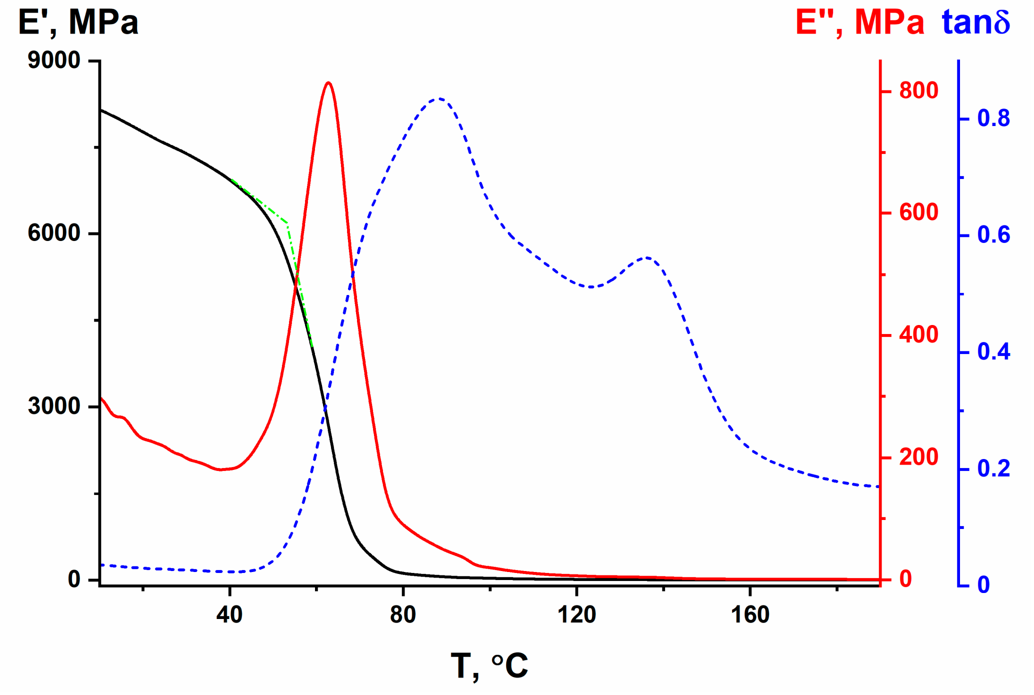

Figure S10. Dynamic mechanical analysis of Co-Copolymer 3: black line – elastic modulus, red line – loss modulus, blue dash – loss tangent, green dash-dot – tangents for glass transition temperature determination.

Table S1. Glass transition temperatures of Co-Copolymers 1-3 obtained from dynamic mechanical analysis.

**Calculation method**	**Copolymers**		
	Co-Copolymer 1	Co-Copolymer 2	Co-Copolymer 3
Tg (onset E′)	75	56	53
Tg (inflection E′)	84	73	67

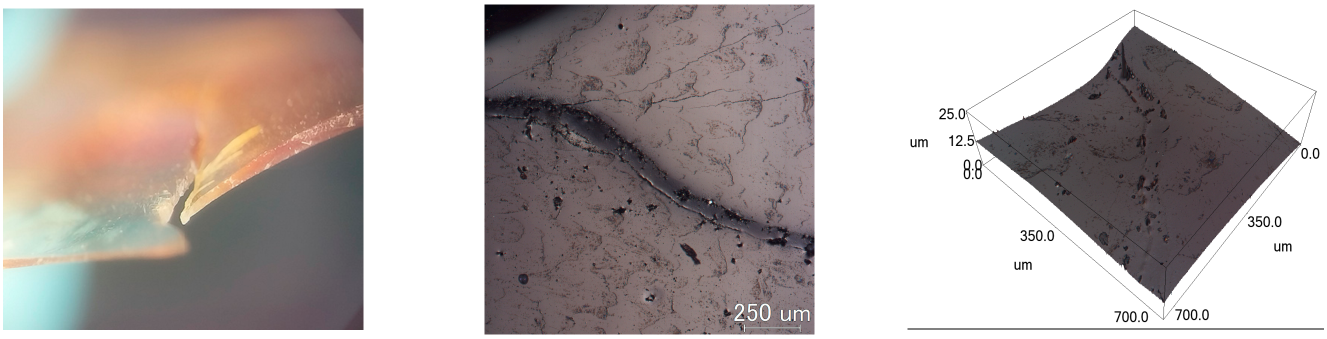

(a) **(b)** **(c)**

Figure S11. Autonomous intrinsic healing of Co-Copolymer 1 - optical microscopy images (**a**) before healing; (**b**) laser scanning microscope image of the crack area after healing, and (**c**) confocal image of its surface.

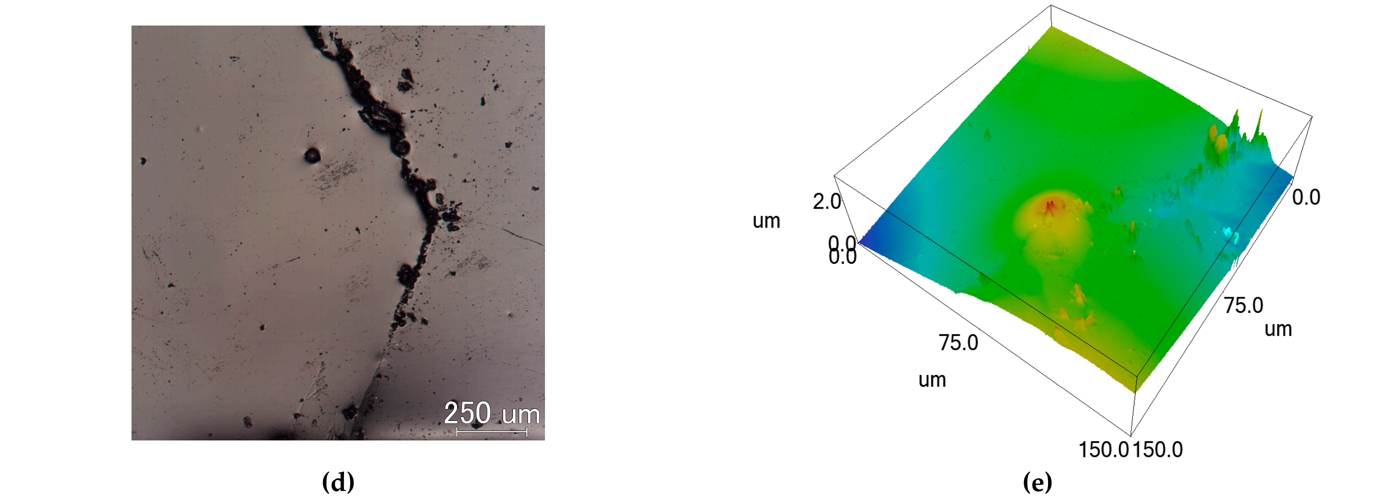

Figure S12. Autonomous intrinsic healing of Co-Copolymer 2 - optical microscopy images (**a-b**) before and (**c**) after healing; (**d**) laser scanning microscope image of the crack area after healing and (**e**) confocal image of its surface.

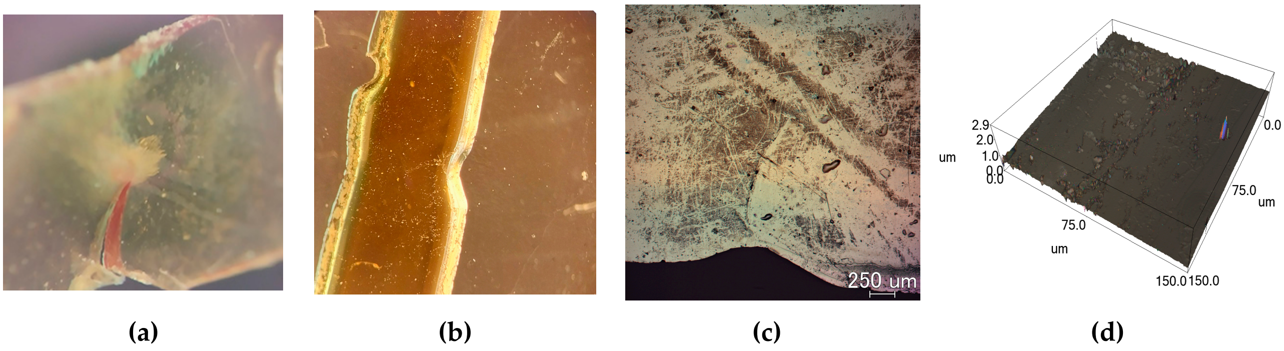

Figure S13. Autonomous intrinsic healing of Co-Copolymer 3 - optical microscopy images (**a**) before and (**b**) after healing; (**c**) laser scanning microscope image of the fracture area after healing and (**d**) confocal image of its surface.

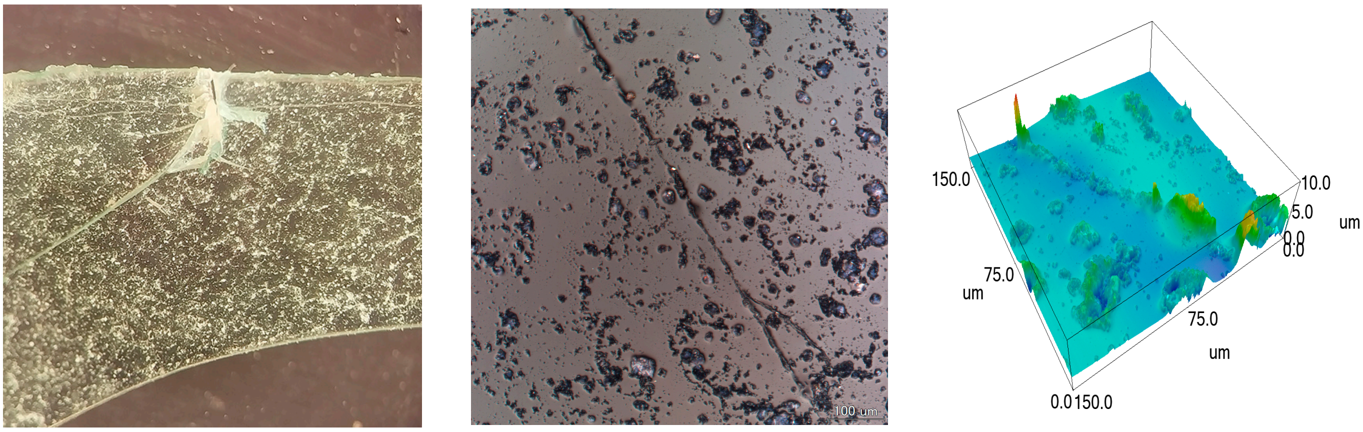

(a) (b) (c)

Figure S14. Autonomous intrinsic healing of Cu-Copolymer 1 - optical microscopy images (**a**) before healing; (**b**) laser scanning microscope image of the crack area after healing, and (**c**) confocal image of its surface.

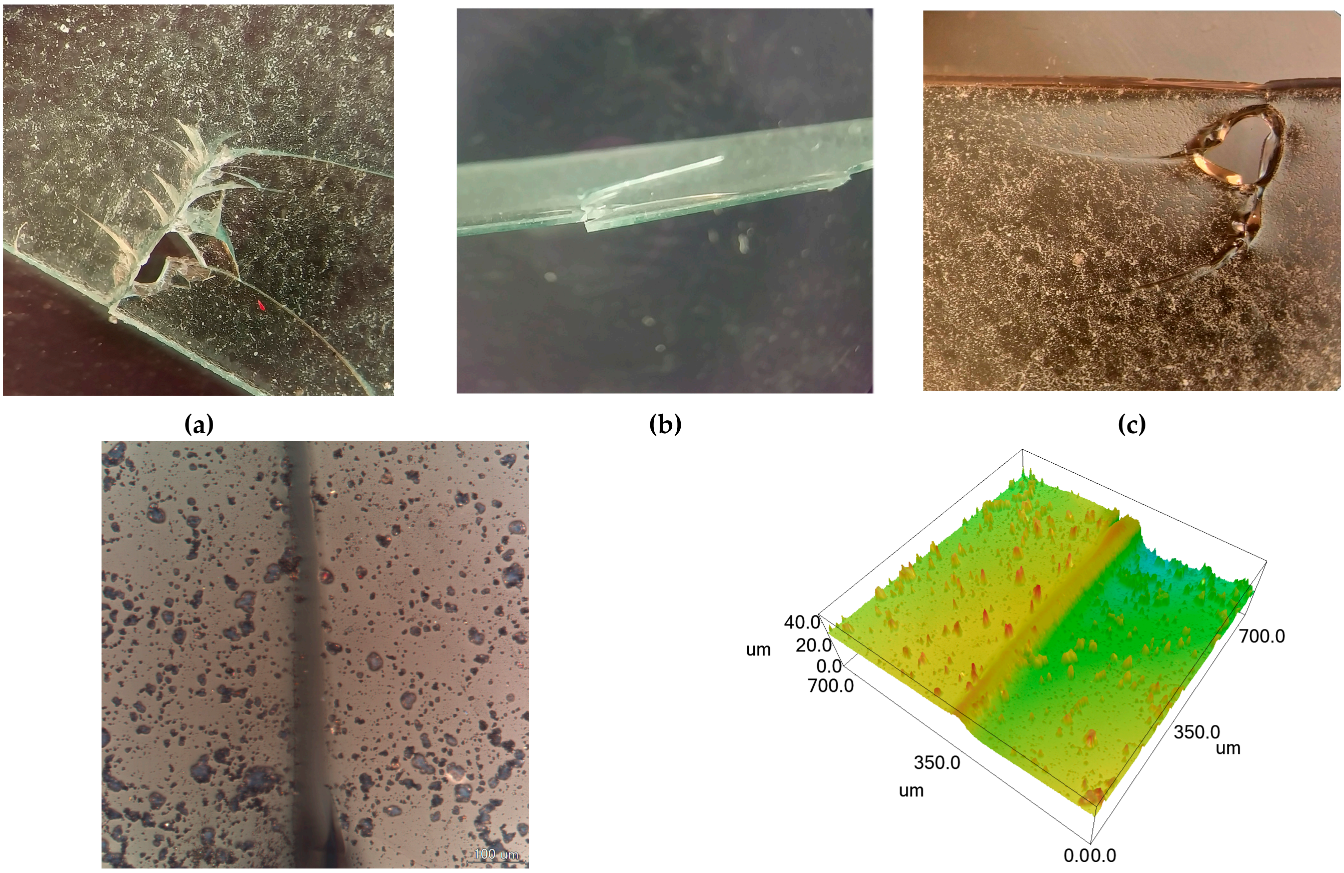

(a) (b) (c)

(d) (e)

Figure S15. Autonomous intrinsic healing of Cu-Copolymer 3 - optical microscopy images (**a-b**) before and (**c**) after healing; (**d**) laser scanning microscope image of the crack area after healing and (**e**) confocal image of its surface.

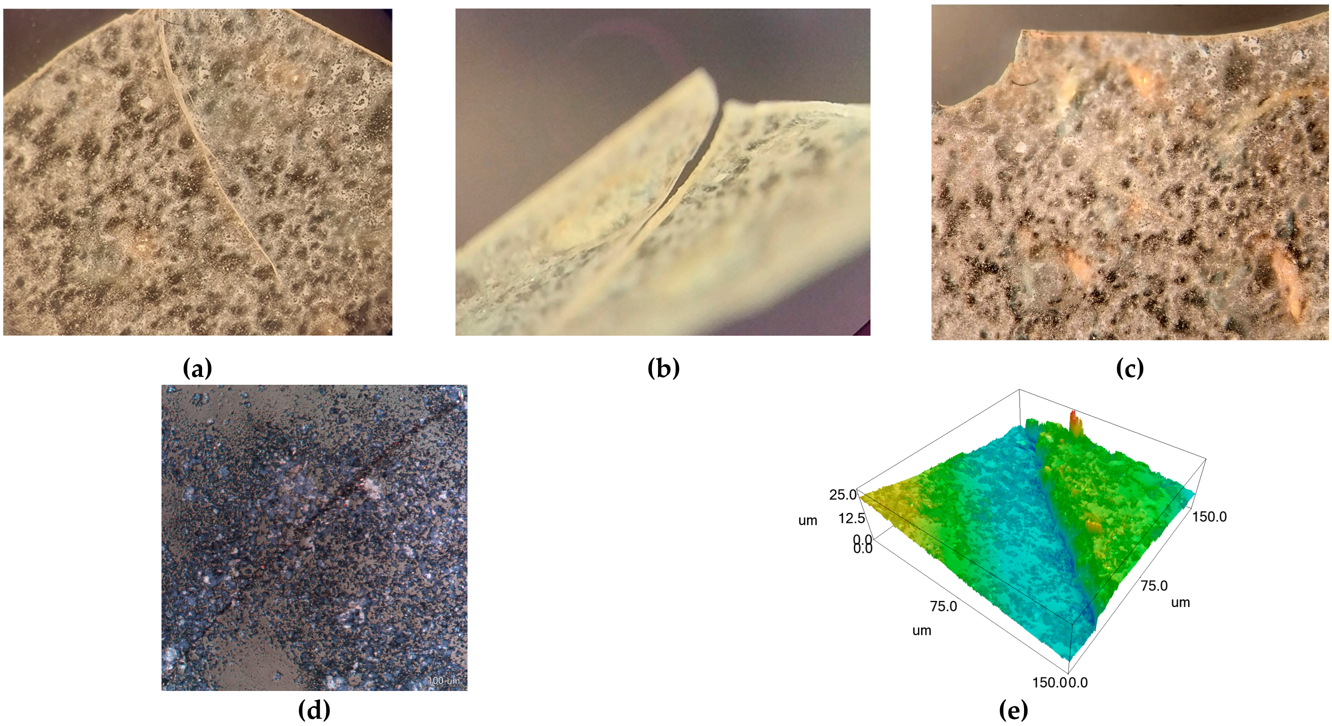

Figure S16. Autonomous intrinsic healing of Ni-Copolymer 1 - optical microscopy images (**a-b**) before and (**c**) after healing; (**d**) laser scanning microscope image of the crack area after healing and (**e**) confocal image of its surface.

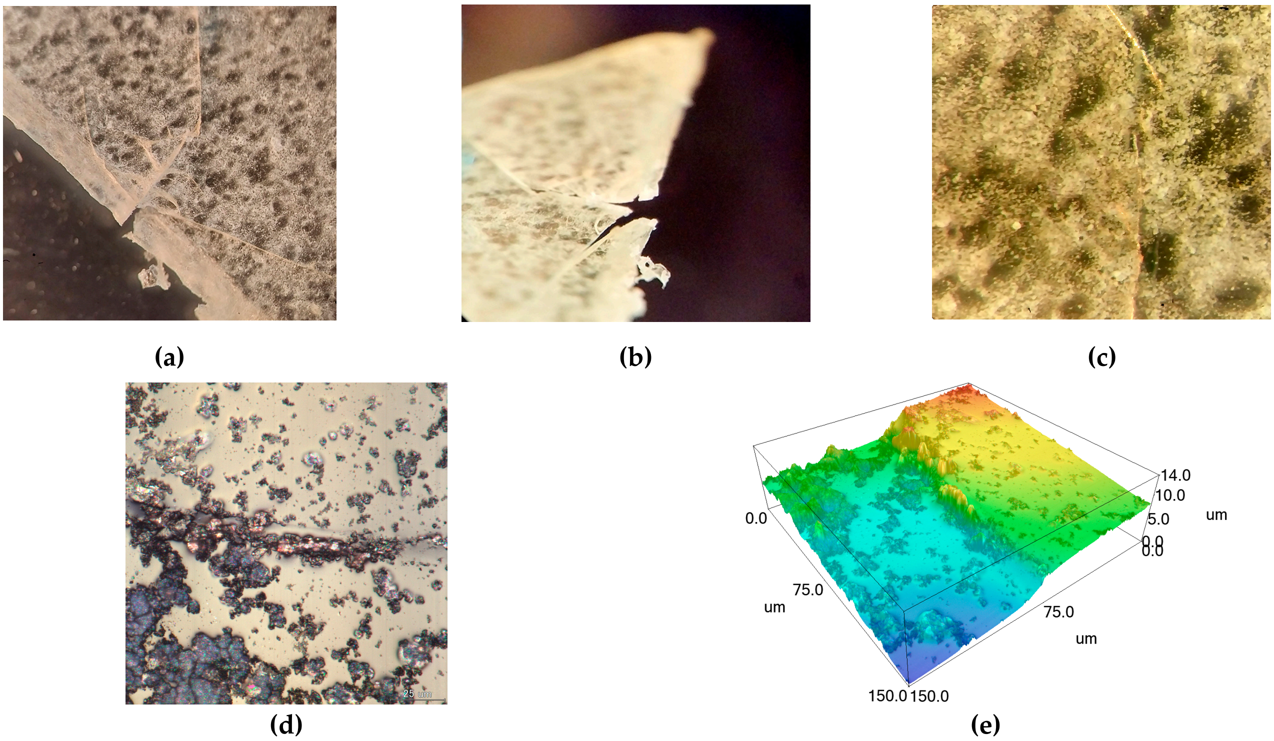

Figure S17. Autonomous intrinsic healing of Ni-Copolymer 2 – optical microscopy images (**a-b**) before and (**c**) after healing; (**d**) laser scanning microscope image of the crack area after healing and (**e**) confocal image of its surface.

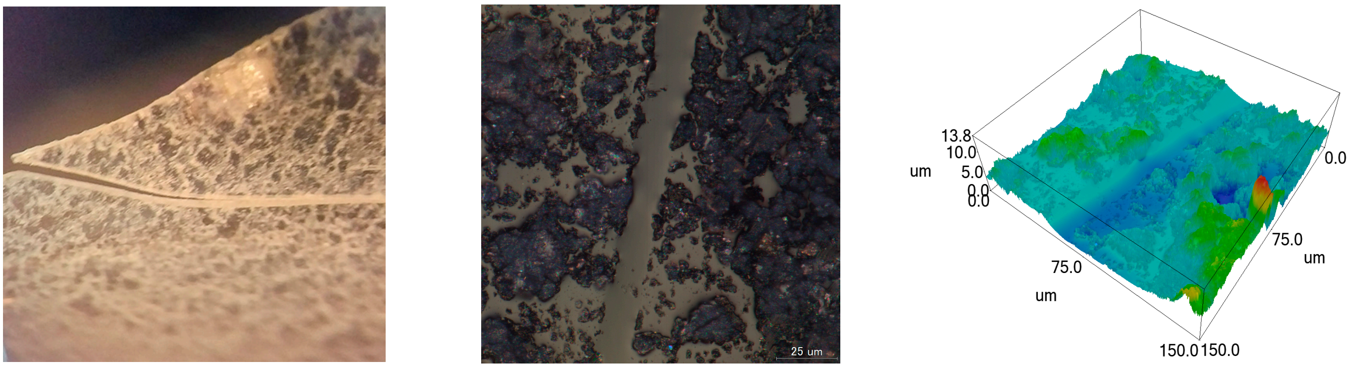

(a) **(b)** **(c)**

Figure S18. Autonomous intrinsic healing of Ni-Copolymer 3 – optical microscopy images (**a**) before healing; (**b**) laser scanning microscope image of the crack area after healing, and (**c**) confocal image of its surface.

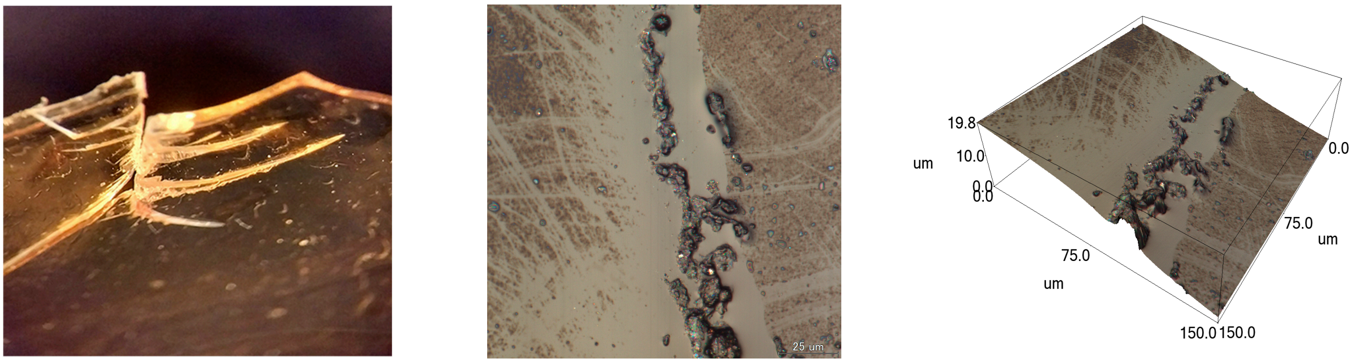

(a) **(b)** **(c)**

Figure S19. Autonomous intrinsic healing of CoA-Copolymer 2 - optical microscopy images (**a**) before healing; (**b**) laser scanning microscope image of the crack area after healing, and (**c**) confocal image of its surface.

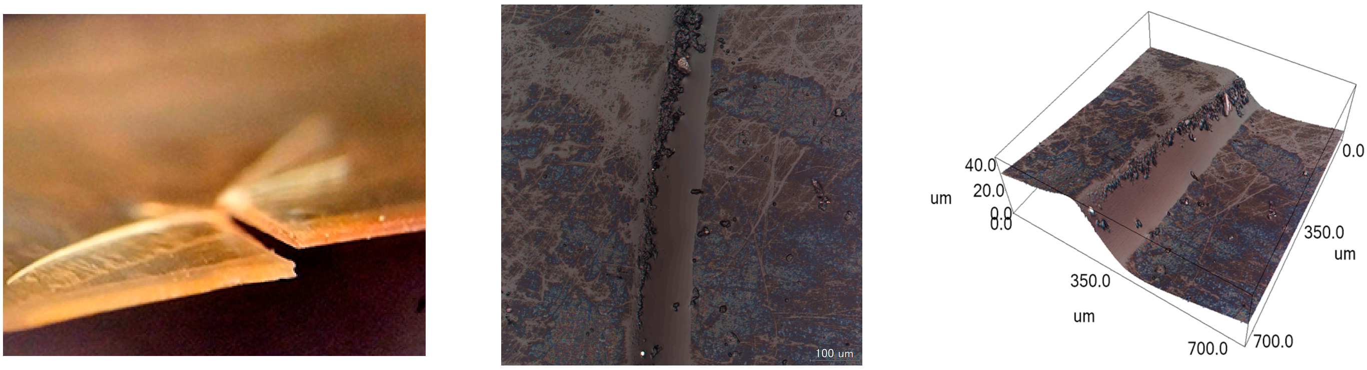

(a) **(b)** **(c)**

Figure S20. Autonomous intrinsic healing of CoA-Copolymer 3 - optical microscopy images (**a**) before healing; (**b**) laser scanning microscope image of the crack area after healing, and (**c**) confocal image of its surface.
